# Geometry of the Proximal Phalanx of Hallux and First Metatarsal Bone to Predict Hallux Abducto Valgus: A Radiological Study

**DOI:** 10.1371/journal.pone.0166197

**Published:** 2016-11-18

**Authors:** Eduardo Perez Boal, Ricardo Becerro de Bengoa Vallejo, Miguel Fuentes Rodriguez, Daniel Lopez Lopez, Marta Elena Losa Iglesias

**Affiliations:** 1 Facultad de Ciencias de la Salud, Universidad Rey Juan Carlos, Alcorcón, Madrid, Spain; 2 Facultad de Enfermería, Fisioterapia y Podologia, Universidad Complutense de Madrid, Madrid, Spain; 3 Research, Health and Podiatry Unit, Department of Health Sciences, Faculty of Nursing and Podiatry, Universidade da Coruña, Coruña, Spain; University of Umeå, SWEDEN

## Abstract

**Background:**

Hallux abducto valgus (HAV) is one of the most common forefoot deformities in adulthood with a variable prevalence but has been reported as high as 48%. The study proposed that HAV development involves a skeletal parameter of the first metatarsal bone and proximal phalanx hallux (PPH) to determine if the length measurements of the metatarsal and PPH can be used to infer adult HAV.

**Methods:**

All consecutive patients over 21 years of age with HAV by roentgenographic evaluation were included in a cross-sectional study. The control group included patients without HAV. The study included 160 individuals. We identified and assessed the following radiographic measurements to evaluate HAV: the distances from the medial (LDM), central (LDC), and lateral (LDL) aspects of the base to the corresponding regions of the head of the PPH. The difference between the medial and lateral aspect of PPH was also calculated.

**Results:**

The reliability of the variables measured in 40 radiographic films show perfect reliability ranging from 0.941 to 1 with a small error ranging from 0.762 to 0. Also, there were no systematic errors between the two measurements for any variable (P > 0.05). The LDM PPH showed the highest reliability and lowest error.

**Conclusion:**

It is more suitable to measure the LDM PPH instead of the LDC PPH when calculating the hallux valgus angle based on our reliability results. When the differences of the medial and lateral PPH are greater, the risk for developing HAV increases.

## Introduction

Hallux abducto valgus (HAV) is one of the most common forefoot deformities in adulthood with a variable prevalence but has been reported as high as 48% [1]. The deformity is most common in women during their fourth, fifth, and sixth decade of life [2] and is usually progressive [3,4], occurring more frequently in elderly women [5]. HAV is considered a deformity of multifactorial origin, primarily attributed to the use of footwear [6,7], genetics [8], and gender [9]. Additional factors also include metatarsus varus [10], abnormal metatarsus length [11] abnormal shape of the metatarsal head [12], action of the foot muscles [13], and foot pronation [14]. Excessive length of the first metatarsal with respect to the second [15–17], also called protrusion [10,11,18,19], has been associated with hallux valgus, but a short first metatarsal relative to the second [20] has also been suggested as an etiologic factor in HAV. Other reported etiologic factors include [19], a high intermetatarsal angle [10,15], and hypermobility [14,21,22].

The association of an excessive absolute length of the first metatarsal and the deformity of hallux valgus has been reported previously [17]. However, there are also authors who state that in feet with this deformity, the first metatarsal is not longer than the second, rather it has a greater relative protrusion [11]. Also, the alteration in the length of the hallux has been related with the etiology of hallux valgus, specifically an excessive length [15,17,23–26].

Typically, measurement of the longitudinal axis of the first metatarsal bone and the proximal phalanx are used to determinate the HAV angle [15,17,27–30]. Tanaka et al [17] reported that, on average, the great toe and first metatarsal of 64 female patients who had hallux valgus were longer than those of normal subjects. However, the length at the tip of the great toe and at the end of the first metatarsal were larger only in the patients who were less than 20 years old, because of the progressive angulation of the great toe and the first metatarsal in the older patients.

Ferrari et al. [31] used a three-dimensional laser scan to measure bone size and shape of the talus, navicular, medial cuneiform, and first metatarsal bones from 107 skeletons of known age and sex, suggesting that the female foot has an underlying anatomical predisposition to first metatarsal adduction and, thus, HAV formation.

Recently, in a cadaveric study [32,33], the dimensions of the proximal phalanx hallux (PPH) in normal feet in both men and women were studied to determinate the height, base width, longitudinal distance of the medial, central and lateral aspect of the proximal phalanx. Significant differences were found between the genders in most dimensions of the PPH, except for the depth of the concave area of the base of the proximal phalanx.

Based on the geometric differences of the first metatarsal bone and PPH, we designed a new approach to radiographically measure this deformity at the medial, central and lateral aspects at the first metatarsal bone and the proximal phalanx in normal and HAV persons to determine any differences. Despite a large number of studies on this subject, the underlying cause of this deformity remains unclear. This divergence of opinions regarding the etiology of HAV underscores the need for a new perspective in order to elucidate the underlying possible etiological factors of this deformity. Unlike previous studies, the current study proposed that HAV development involves a skeletal parameter of the first metatarsal bone and PPH to determine if the length measurements of the metatarsal and PPH can be used to infer adult HAV. We hypothesized that the length of lateral, medial and central sides of the first metatarsal bone and the first proximal phalanx were different depending on if a person had hallux valgus. Further, a gender-specific effect on the first metatarsal bone and/or PPH was also evaluated to understand if women were predisposed developing HAV greater than men.

## Material and Methods

Prospective longitudinal case control study design. The subjects of this study were patients attending the foot and ankle clinic at the CEMTRO hospital of Madrid from January 20013 to January 2015. This study has been approved by the Experimental Ethics Committee of the University of Rey Juan Carlos, and written consent was obtained for all participants.

All consecutive patients over 21 years of age with HAV by roentgenographic evaluation were included in a cross-sectional study. The control group included patients without HAV who visited the clinic for other orthopedic conditions of the foot. Controls were matched to cases according to age and gender. Exclusion criteria included previous foot surgery, malformations on the lower extremity, foot trauma, and neurological diseases. The study included 160 individuals (Table 1).

**Table 1:** Demographic data of the study population.

Group	All subjects		Males		Females		P value
	Age: mean±SD (95% CI)	N feet (R/L)	Age: mean±SD (95% CI)	N feet (R/L)	Age: mean±SD (95% CI)	N feet (R/L)	
**Total population (N = 160)**	49.80 ± 13.71 (47.55–52.05)	160 (104/107)	48.74 ± 15.18 (45.28–52.21)	85 (54/55)	50.93 ± 11.93 (48.11–53.75)	75 (50/52)	0.244
**Control group (N = 81)**	49.60 ± 13.89 (40.02–59.18)	81 (52/59)	46.72 ± 15.75 (42.07–51.37)	44 (28/31)	52.86 ± 10.67 (49.42–56.29)	37 (24/28)	0.017
**HAV group (N = 79)**	50.02 ± 13.58 (47.02–53.01)	79 (52/48)	51.12 ± 14.26 (46.75–55.48)	41 (26/24)	48.92 ± 12.91 (44.82–53.02)	38 (26/24)	0.420

Abbreviations: N, sample size; SD, standard deviation. R, Right; L, left. P value determined with independent t-tests. P<0.01 considered statistically significant.

The diagnosis of HAV was based on the clinical appearance of the forefoot and on radiographic evaluation [3,10,34–36] under standardized weight-bearing conditions [37]. The diagnostic angle for HAV is 15° between the longitudinal axis of the first metatarsal and that of the proximal phalanx [38].

The foot radiographs were taken using a General Electrics Discovery XR656 Plus (General Electrics Research, Milwaukee, WI) at a source-to-image distance of 100 cm and were set to 60 kVp and 2,5 mAs with the patient standing. We retrieved the radiographic images using a picture archiving and communication system (PACS) (IMPAX; Agfa Healthcare, Mortsel, Belgium), and radiographic measurements were performed using PACS software and a digital radiographic imaging and measuring system (AutoCad 2013, Autodesk Inc., San Rafael, CA)

Dorsoplantar radiographs for weight-bearing conditions were performed with the patients standing on both feet with the knee extended. The medial border of the foot was aligned to avoid internal or external rotation of the leg and the foot was pointed straight forward in neutral rotation, parallel to the medial sagittal plane. The X-ray beam was inclined 15° in an anterior-posterior direction centered on the second tarsometatarsal joint at a distance of 100 cm [37,39–42].

**Fig 1:**
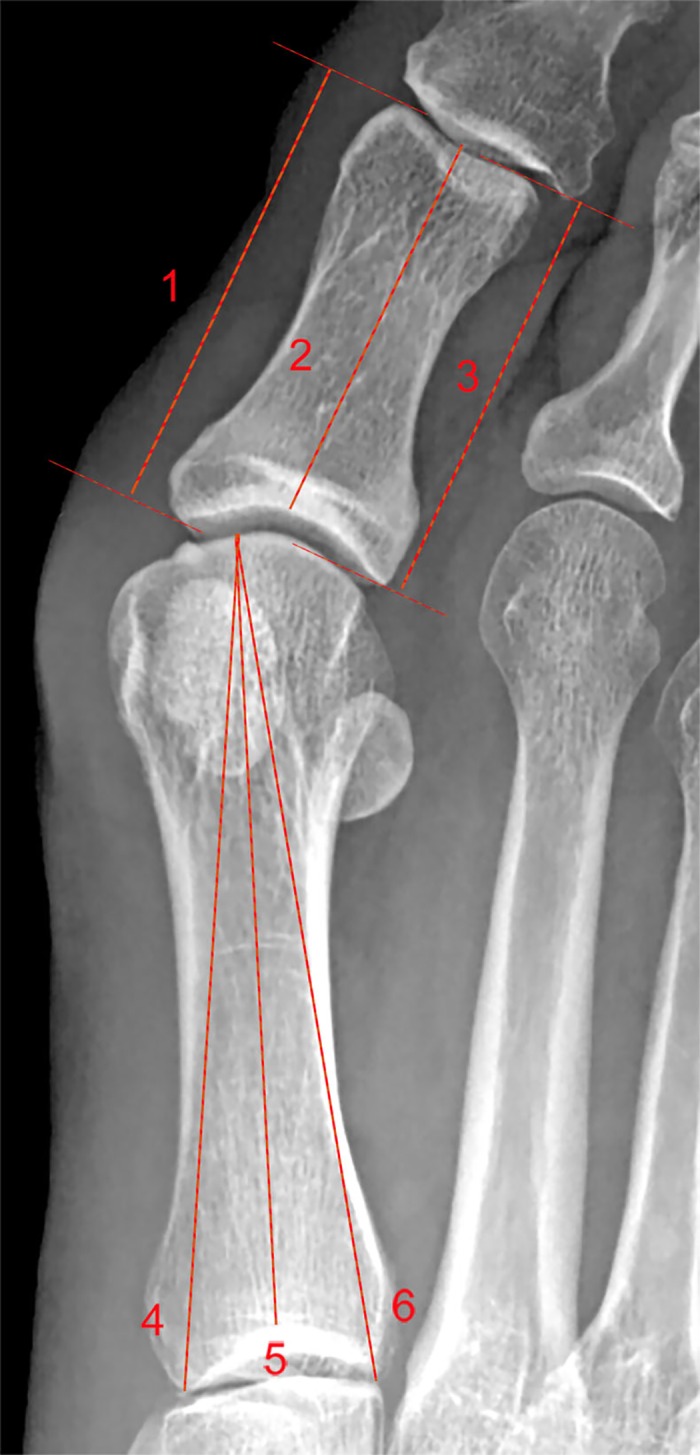
On the weight-bearing dorsoplantar foot radiograph, lines were measured and drawn; line 1 is the longitudinal distance medial aspect of the proximal phalanx (LDMPPH); line 2 is the longitudinal distance central aspect of the proximal phalanx (LDCPPH); line 3 is the longitudinal distance lateral aspect of the proximal phalanx (LDLPPH); line 4 is the longitudinal distance medial aspect of the I metatarsal bone (LDM-M); line 5 is the longitudinal distance central aspect of the I metatarsal bone (LDC-M); line 6 is the longitudinal distance lateral aspect of the I metatarsal bone (LDL-M).

We identified and assessed the following radiographic measurements to evaluate HAV: the distances from the medial (LDM), central (LDC), and lateral (LDL) aspects of the base to the corresponding regions of the head of the PPH [32]. The difference between the medial and lateral aspect of PPH was also calculated [33] (Fig 1). All measurements were performed in an independent blinded fashion.

### Statistical analyses

To determine the reliability of the measurement procedure, 20 feet from the control group and 20 feet from the HAV group were chosen at random. Measurements were made with an interval of five days between the first and second measurements. The intraclass coefficient of this correlation was calculated according to the methodology used previously by Shrout and Fleiss [43].

The minimum number of patients required was calculated based on reliability testing to determine reliability. In this study, the ICCs were used for reliability testing at a target value of 0.8 and a 95% CI of 0.2. We calculated the sample size to be 36 patients with a Bonett’s approximation [44]. One foot from each patient was selected by random sampling for statistical independence and included for data analysis [45]. We calculated intraobserver reliabilities using the ICCs; 95% CIs were determined in the setting of a two-way random effect model, a single measurement, and absolute agreement. Radiographic variables were measured on the 36 films, and the data were used to determine if the measurements were reliable prior to completing measurements for the entire study population [46].

Measurements included 211 radiographs from 109 feet of men (59 in control group, 50 in HAV group) and 102 feet of women (52 in control group, 50 in HAV group). The paired t test was used to compare the mean values between the first and second measurements. Intraclass correlation coefficients (ICCs) were calculated to first determine reliability between trials. The average of two trials for each test session on each radiograph was used to calculate intersession reliability using an ICC[1,k] model.

The ICC measures the relative error, the degree to which individuals maintain their position or value in repeated measurements [43,47]. The ICC ranges from 0 to 1[25]. To analyze the results we used the criteria of Landis and Koch (1977), who consider that an ICC value from 0.40 to less than 0.60 has moderate reliability, from 0.60 to less than 0.80 is reliable, and from 0.81 or greater, is considered almost perfect [48]. The SEM is a measure of absolute reliability; the lower the SEM, the greater reliability [49,50]. The Kolmogorov-Smirnov test was used to evaluate the normality of the data. The results indicated that the data were normally distributed and that parametric statistical tests were most appropriate.

Age, gender and descriptive data of variables were summarized as mean ± standard deviation (SD) and 95% confidence interval. To determine differences between groups and gender, the independent t-test was used. Analysis of variance (ANOVA) was performed to determine whether there were statistically significant differences between groups and gender in the LDM, LDC, and LDL of the PPH and first metatarsal bone. The dependent variables were the presence or absence of HAV deformity and the independent variables were the lateral, medial and central aspect length of PPH, lateral, medial and central aspect length of I MTT. Pearson’s correlation test was applied to the data in order to determine whether there was any association among the radiographic measurements between measured variables compared with HAV angle and HAV group.

In all of the analyses, statistical significance was established by a P value of less than 0.01, with an interval of confidence of 99%, and analyses were performed with commercially available software (SPSS 19.0, SPSS Inc, Chicago, Illinois). All data analyzed for this paper are publicly archived at Figshare (https://dx.doi.org/10.6084/m9.figshare.4109631).

## Results

The demographics data are shown in Table 1.

The reliability of the variables measured in 40 radiographic films are shown in Table 2, and all show perfect reliability ranging from 0.941 to 1 with a small error ranging from 0.762 to 0. Also, there were no systematic errors between the two measurements for any variable (P > 0.05). The LDM PPH showed the highest reliability and lowest error, and in the metatarsal bone the LDC M showed the highest reliability and lowest error.

**Table 2:** Reliability of variables from the first and second sessions.

	First measurement	Second measurement	Reliability		Kolmogorov-Smirnov	Systematic error between first and second session
Variable N = 40	Mean ±SD	Mean ±SD	ICC 95% IC (Li-Ls)	SEM	P value	P value
	(min-max)	(min-max)				
LDM PPH	35.60 ± 3.22 (28.32–41.87)	35.59 ± 3.23 (28.30–42.04)	1.00(1.00–1.00)	0	0.07	0.744
LDC PPH	29.95 ± 2.95 (23.79–35.25)	29.78 ± 3.31 (20.24–35.38)	0.941(0.888.0.969)	0.762	0.052	0.488
LDL PPH	32.67 ± 3.30 (26.03–37.94)	32.59 ± 3.31 (26.02–38.03)	0.998(0.993–0.998)	0.147	0.2	0.178
DIF. LDM-LDL (PPH)	2.92 ± 1.15	3.00 ± 1.10	0.972(0.946–0.985)	0.189	0.056	0.174
LDM M	67.35 ± 5.12 (57.29–75.22)	67.38 ±5.12 (57.19–75.46)	0.999(0.998–1.00)	0.162	0.2	0.573
LDC M	65.38 ± 5.62 (54.62–74.40)	65.44 ± 5.58 (54.77–74.56)	1.00(1.00–1.00)	0	0.2	0.051
LDL M	67.86 ± 5.46 (57.27–76.74)	67.94 ±5.45 (57.05–76.86)	0.999(0.998–1.00)	0.172	0.085	0.111

Abbreviations: N, sample size; SD, standard deviation; Min, minimum value; Max, maximum value; LDM PPH, longitudinal distance of the medial aspect of hallux; LDC PPH, longitudinal distance of the central aspect of hallux; LDL PPH, longitudinal distance of the lateral aspect of hallux; DIF LDM–LDL (PPH), Difference between Longitudinal distance of Medial aspect and Longitudinal distance of Lateral aspect of Hallux; LDM M, longitudinal distance of the medial aspect of first metatarsal; LDC M, longitudinal distance of the central aspect of first metatarsal; LDL M, longitudinal distance of the lateral aspect of first metatarsal; SD, standard deviation. P < 0.05 (with a 95% confidence interval) was considered statistically significant. SD, Standard deviation. ICC, Intraclass Correlation Coefficient; Li, inferior limit; Ls, Superior limit; SEM, standard error of the mean. P < 0.01 (with a 99% confidence interval) was considered statistically significant.

Based on the strong reliability, the remaining radiographs were analyzed (total of 211 feet; Table 1). The only variable with a significant correlation with HAV in females was DIF LDM-LDL (PPH) (r = 0.279, p = 0.048). The geometry of the PPH in the control group between males and females showed significant medial and central longitudinal distances (P < 0.01). The geometry of the first metatarsal bone was significantly shorter for females at medial, central and lateral longitudinal distances (P < 0.001; Table 3). The geometry of PPH in the HAV group showed a significantly smaller PPH in females versus males (P < 0.01), but no difference in the first metatarsal between the genders (P > 0.05; Table 3).

**Table 3:** Comparative measurements of the proximal phalanx and first metatarsal bone.

	CONTROL GROUP				HAV GROUP			
	Total Sample (N = 81)	Male (N = 44)	Female (N = 37)	P value	Total Sample (N = 79)	Male (N = 41)	Female (N = 38)	P value
Variable (mm)	Mean ± SD (range)	Mean ± SD (range)	Mean ± SD (range)	Male vs. Female	Mean ± SD (range)	Mean ± SD (range)	Mean ± SD (range)	Male vs. Female
LDM PPH	35.58 ± 3.37 (27.44–45.17)	37.08 ± 2.97 (28.40–45.17)	33.89 ± 2.99 (27.44–41.73)	0.020	36.08 ± 3.37 (28.34–44.40)	36.97 ± 2.87 (31.36–44.40)	35.18 ± 3.61 (28.34–43.09)	0.007
LDC PPH	30.60 ± 30.40 (20.18–40.44)	32.04 ± 2.99 (23.99–40.44)	28.97 ± 3.13 (20.18–36.81)	0.001	29.83 ± 3.12 (28.76–39.35)	30.71 ± 2.84 (24.40–37.63)	28.94 ± 3.16 (23.14–36.20)	0.004
LDL PPH	33.46 ± 3.42 (24.99–43.99)	34.95 ± 3.10 (26.21–43.99)	31.77 ± 2.98 (24.99–39.62)	0.105	32.50 ± 3.23 (25.31–40.39)	33.47 ± 2.88 (28.49–40.39)	31.53 ± 3.29 (25.31–38.86)	0.002
DIF. LDM-LDL (PPH)	2.12 ± 0.46 (0.75–3.14)	2.13 ± 0.49 (0.75–3.14)	2.21 ± 0.45 (1.33–3.13)	0.930	3.57 ± 0.78 (1.39–5.74)	3.50 ± 0.68 (1.39–4.82)	3.65 ± 0.87 (2.27–5.74)	0.327
LDM M	66.41 ± 5.42 (54.19–78.83)	68.80 ± 4.39 (56.73–78.83)	63.70 ± 5.25 (54.19–73.86)	0.001	68.84 ± 6.38 (54.05–84.12)	69.68 ± 6.50 (57.60–81.65)	68.00 ± 6.19 (54.05–84.12)	0.188
LDC M	64.25 ± 5.54 (50.50–76.47)	66.44 ± 4.81 (53.41–76.47)	61.77 ± 5.31 (50.50–71.20)	0.001	67.36 ± 6.44 (53.33–82.74)	67.71 ± 6.74 (55.26–79.34)	67.01 ± 6.18 (53.33–82.74)	0.591
LDL M	66.64 ± 5.70 (53.60–78.81)	68.94 ± 4.78 (57.26–78.81)	64.03 ± 5.58 (53.60–75.73)	0.001	70.24 ± 6.25 (56.89–84.60)	71.11 ± 6.40 (59.7–84.6)	69.36 ± 6.03 (56.89–8.51)	0.163

Abbreviations: LDM PPH, longitudinal distance of the medial aspect of hallux; LDC PPH, longitudinal distance of the central aspect of hallux; LDL PPH, longitudinal distance of the lateral aspect of hallux; DIF LDM–LDL (PPH), Difference between Longitudinal distance of Medial aspect and Longitudinal distance of Lateral aspect of Hallux; LDM M, longitudinal distance of the medial aspect of first metatarsal; LDC M, longitudinal distance of the central aspect of first metatarsal; LDL M, longitudinal distance of the lateral aspect of first metatarsal; SD, standard deviation. P < 0.01 (with a 99% confidence interval) was considered statistically significant.

The male group showed significantly shorter LDC PPH (P < 0.001) and LDL PPH (P = 0.005) in the HAV group, but no differences with regard to the LDM HHP The LDM–LDL (PPH) was significantly greater in males with HAV(3.50 ± 0.68 mm) compared to controls (2.13 ± 0.49 mm). Similar results occurred in the female group, such that LDM PPH in females with HAV were significantly longer than controls (P = 0.025), but there were no differences in the LDC (P = 0.484) and LDL (P = 0.352) for the female HAV group (Table 4). All distances from first metatarsal bone were significantly longer in females with HAV versus controls (P < 0.001; Table 4).

**Table 4:** Comparative measurements of the proximal phalanx and first metatarsal bone.

	Male			Female		
	Control group (N = 44)	HAV group (N = 41)		Control group (N = 37)	HAV group (N = 38)	
Variable (mm)	Mean ± SD (range)	Mean ± SD (range)	P value	Mean ± SD (range)	Mean ± SD (range)	P value
LDM PPH	37.08 ± 2.97 (28.40–45.17)	36.97 ± 2.87 (31.36–44.40)	0.425	33.89 ± 2.99 (27.44–41.73)	35.18 ± 3.61 (28.34–43.09)	0.025
LDC PPH	32.04 ± 2.99 (23.99–40.44)	30.71 ± 2.84 (24.40–37.63)	0.001	28.97 ± 3.13 (20.18–36.81)	28.94 ± 3.16 (23.14–36.20)	0.484
LDL PPH	34.95 ± 3.10 (26.21–43.99)	33.47 ± 2.88 (28.49–40.39)	0.005	31.77 ± 2.98 (24.99–39.62)	31.53 ± 3.29 (25.31–38.86)	0.352
DIF LDM–LDL (PPH)	2.13 ± 0.49 (0.75–3.14)	3.50 ± 0.68 (1.39–4.82)	0.001	2.21 ± 0.45 (1.33–3.13)	3.65 ± 0.87 (2.27–5.74)	0.001
LDM M	68.80 ± 4.39 (56.73–78.83)	69.68 ± 6.50 (57.60–81.65)	0.201	63.70 ± 5.25 (54.19–73.86)	68.00 ± 6.19 (54.05–84.12)	0.001
LDC M	66.44 ± 4.81 (53.41–76.47)	67.71 ± 6.74 (55.26–79.34)	0.126	61.77 ± 5.31 (50.50–71.20)	67.01 ± 6.18 (53.33–82.74)	0.001
LDL M	68.94 ± 4.78 (57.26–78.81)	71.11 ± 6.40 (59.7–84.6)	0.022	64.03 ± 5.58 (53.60–75.73)	69.36 ± 6.03 (56.89–8.51)	0.001

Abbreviations: LDM PPH, longitudinal distance of the medial aspect of hallux; LDC PPH, longitudinal distance of the central aspect of hallux; LDL PPH, longitudinal distance of the lateral aspect of hallux; DIF LDM–LDL (PPH), Difference between Longitudinal distance of Medial aspect and Longitudinal distance of Lateral aspect of Hallux; LDM M, longitudinal distance of the medial aspect of first metatarsal; LDC M, longitudinal distance of the central aspect of first metatarsal; LDL M, longitudinal distance of the lateral aspect of first metatarsal; SD, standard deviation. P < 0.01 (with a 99% confidence interval) was considered statistically significant.

Similar results occurred in the female group, such that LDM PPH in females with HAV were significantly longer than controls at 95% IC (P = 0.025), but there were no differences in the LDC (P = 0.484) and LDL (P = 0.352) for the female HAV group (Table 4).

**Fig 2:**
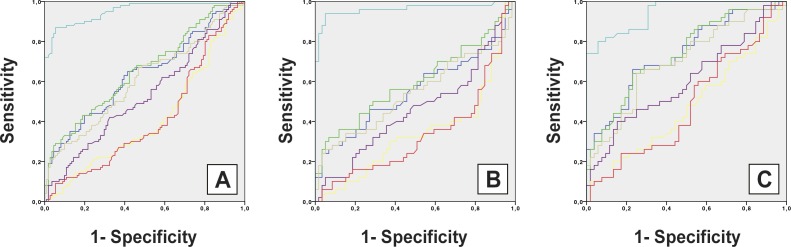
The capacity of variables for detecting presence of Hallux Abductus Valgus in the total population (A), males (B), and females (C). Abbreviations: LDM PPH, longitudinal distance of the medial aspect of hallux (purple line); LDC PPH, longitudinal distance of the central aspect of hallux (red line); LDL PPH, longitudinal distance of the lateral aspect of hallux (yellow line); DIF LDM–LDL (PPH), Difference between longitudinal distance of medial aspect and Longitudinal distance of lateral aspect of hallux (light blue line); LDM M, longitudinal distance of the medial aspect of first metatarsal (olive green line); LDC M, longitudinal distance of the central aspect of first metatarsal (dark blue line); LDL M, longitudinal distance of the lateral aspect of first metatarsal (dark green line); SD, standard deviation. P < 0.01 (with a 99% confidence interval) was considered statistically significant.

Surprisingly, there were significant differences with regard to the geometry of the PPH calculated or according to variable DIF LDM–LDL (PPH) in predicting the presence of HAV in our study population (Table 5). Cutoff values of the variable DIF LDM–LDL (PPH) defined at relatively greater differences distances of medial and lateral aspects of the PPH were very sensitive for detecting the presence of HAV and also very specific for predicting a lack of HAV. Differences between genders were determined between the optimal DIF LDM–LDL (PPH) cutoff values to predict presence of HAV, as determined by a balance of sensitivity and specificity. The area under the ROC curve was significantly greater P < 0.01) for presence of HAV when DIF LDM–LDL (PPH) in the total population were ≥ 2.81 mm (Fig 2A), in males were ≥ 2.81 mm (Fig 2B), and in females were ≥ 2.9 mm (Fig 2C). Conversely, cutoff values made at lower DIF LDM–LDL (PPH) were sensitive for predicting absence of HAV.

**Table 5:** Optimal Cut-off Value to Predict Hallux Abductus Valgus.

POPULATION	VARIABLE	Optimal cutoff value (mm) HAV presence	AREA UNDER THE ROC CURVE (95% CI)	P VALUE	SENSIVITY %	SPECIFICITY %
TOTAL N = 211 (HAV Group N = 100; Control Group N = 111)	LDM PPH	≥ 36,945	0.530(0.452–0.609)	0,445	42	67,6
	LDC PPH	≥ 30,325	0.633(0.556–0.713)	0,001	12	88,3
	LDL PPH	≥ 33,540	0.407(0.330–0.485)	0,020	35	42,3
	LDM M	≥ 66,315	0.607(0.531–0.684)	0,007	67	53,2
	LDC M	≥ 65,605	0.633(0.557–0.708)	0,001	65	58,6
	LDL M	≥ 72,105	0.655(0.581–0.728)	0,001	43	81,1
	DIF LDM-LDL PPH	≥ 2,810	0.952(0.924–0.980)	0,001	87	94,6
MALE N = 109 (HAV Group N = 50; Control Group N = 59)	LDM PPH	≥ 35,955	0.460(0.349–0.571)	0,477	58	23,7
	LDC PPH	≥ 30,780	0.338(0.232–0.443)	0,004	42	22
	LDL PPH	≥ 33,660	0.343(0.238–0.449)	0,005	42	18,6
	LDM M	≥ 75,005	0.543(0.430–0.656)	0,440	26	94,9
	LDC M	≥ 73,995	0.552(0.439–0.665)	0,351	24	96,6
	LDL M	≥ 74,070	0.597(0.486–0.707)	0,083	32	93,2
	DIF LDM-LDL PPH	≥ 2,810	0.962(0.921–1.00)	0,001	94	94,9
FEMALE N = 102 (HAV Group N = 50; Control Group N = 52)	LDM PPH	≥ 36,225	0.602(0.491–0.712)	0,077	40	84,6
	LDC PPH	≥ 29,890	0.482(0.368–0.595)	0,753	30	53,8
	LDL PPH	≥ 29,260	0.473(0.359–0.586)	0,632	74	15,4
	LDM M	≥ 66,315	0.699(0.597–0.800)	0,001	64	75
	LDC M	≥ 65,605	0.735(0.639–0.831)	0,001	66	76,9
	LDL M	≥ 67,925	0.738(0.642–0.833)	0,001	64	76,9
	DIF LDM-LDL PPH	≥ 2,900	0.944(0.904–0.983)	0,001	80	94,2

Abbreviations: ROC, receiver operating characteristic; CI, confidence interval

## Discussion

Traditionally, the hallux valgus angle as measured on radiographs is used as the gold standard to determine hallux valgus deformity [36]. This is despite the fact that hallux valgus is a complex three-dimensional deformity, and the hallux valgus angle can only explain the deformity in one plane. Here, we present a prospective longitudinal case control study that compares the geometry of the PPH and first metatarsal bone in patients with and without HAV deformity. We investigated the reliabilities of radiographic measurements of the longitudinal axis of PPH and the first metatarsal bone to determine which axis or line from radiographic measurements is more reliable to measure the hallux valgus angle. We found the LDM PPH I to be more reliable with an ICC of 1 and inferior and superior limits of 1.00 and 1.00. While the measurement of the longitudinal axis of the PPH, LDC PPH, showed an ICC of 0.941 with inferior and superior limits of 0.888 and 0.969, respectively. Based on these results, we postulate the LDM PPH is a more suitable measurement than the longitudinal axis of the PPH or LDC PPH.

Further, the longitudinal axis of the first metatarsal, LDC M, showed a perfect ICC value with inferior and superior limits of 1.00 and 1.00, respectively. The results show that in patients without HAV, females have shorter LDM PPH and first metatarsal bones than do males. Alternatively, females in HAV group exhibit a significantly smaller PPH than males with HAV. But surprisingly, the length of the first metatarsal bone in females with HAV is much longer in its LDM M, LDC M and LDL M than males with HAV. In males, the longitudinal distances at PPH were significantly shorter for LDL PPH, but similar in length for the LDM PPH. However, the contrary is true for females, as they have significantly longer LDM PPH but similar lengths of the LDL and LDC PPH.

In males with HAV, the LDL M is significantly longer than controls, but the LDM M and LDCM are of similar length. In females with HAV, the LDM M, LDC M and LDL M at PPH are all significantly longer than controls. Also, females with HAV exhibit a significantly longer first metatarsal bone compared to females without HAV, and have similar length as males with HAV.

Traditionally, a measurement of first metatarsal protrusion distance has been used to calculate the length of the first metatarsal bone [10], and it is been reported that a shorter first metatarsal bone relative to the second is associated with HAV deformity [17,20,51].We emphasize that these studies did not take into account the difference between the arcs of the bisecting lines of the first and second metatarsals from the common intersection of the two lines. The first metatarsal bone is actually not shorter; rather it is longer in patients who have HAV, but appears shorter due to its medial displacement, which creates a greater first intermetatarsal angle.

Our results demonstrate that the larger the difference between the LDC and LDL PPH, the greater the possibility to develop an HAV deformity. Importantly, we determined that females have a shorter first metatarsal compared to males, smaller differences of LDM and LDL PPH, indicating a lower possibility to develop HAV. Here, we evaluated a new measurement that has the potential to be used to determine the risk for developing HAV.

### Limitations

Our measurements showed a very small error, but all of the measurements showed almost perfect reliability with ICC´s ranging from 0.94 to 1.00, with no systematic error between the first and second measurements. The hallux valgus angle and geometry of the PPH and metatarsal bone are measured from dorsoplantar radiographic images in a clinical setting. This could introduce an error caused by evaluating a three-dimensional deformity with a two-dimensional radiographic tool. However, this is the same approach used to evaluate HAV in the clinical setting. Perhaps a cadaveric study would clarify this issue. Additionally, all measurements were performed in a Caucasian population, which could potentially influence the results, so increasing the ethnic diversity of the study participants is important to determine geometrical differences. Although radiographs were calibrated using the software, potential limitations of a reliability study could be due to a human error in placing the markers on the X-ray that can influence the readings obtained from the computer measuring software, but this study was performed as in clinical settings.

## Conclusion

We postulate that it is more suitable to measure the LDM PPH instead of the LDC PPH when calculating the hallux valgus angle based on our reliability findings. When the differences of the medial and lateral PPH are greater, the risk for developing HAV increases in males and females.

Therefore, in men in with the morphology of the PPH, the LDL PPH are shorter, producing a difference with the LDM PPH, resulting a DIF LDM-LDL PPH ≥ 2.81mm and a predisposition to a HAV deformity. Females with the shorter LDM PPH resulting a DIF LDM-LDL PPH of ≥ 2.90 mm are predisposed to a HAV deformity. Comparing the total population with and without HAV, when the LDM PPH and LDL PPH results with a DIF LDM-LDL PPH of ≥ 2.81 mm, results in a predisposing risk factor to develop HAV deformity.

Similarly, men with HAV at the first metatarsal have an LDL M longer compared to men without HAV. Further, females with HAV had a longer first metatarsal bone in the three measured aspects (LDM, LDC and DL M) compared to women without HAV. Thus, morphology might be a factor in the formation of HAV, for both men and women.
